# Supplementary material for: A comparison of ‘pruning’ during multi-step planning in depressed and healthy individuals
Source: Psychol Med. 2021 Mar 12;52(16):3948–56. doi: 10.1017/S0033291721000799 (PMC9811346; doi:10.1017/S0033291721000799)
Supplement: Supplementary file 1 [file S0033291721000799sup001.docx]

**Supplementary Materials**

There were no significant or meaningful associations between specific pruning, general pruning, or sensitivities to any of the four transition types and participants’ age (all *p*s > 0.268, all *BF*s < 0.522), years of education (all *p*s > 0.396, all *BF*s > 0.419) or IQ (all *p*s > 0.173, all *BF*s > 0.742).

There was a strong and meaningful difference between the reward sensitivities of the Pruning rho (i.e. ‘winning’) model and the Lookahead rho (i.e. baseline) model, in terms of the -140 transitions (*t*(60) = 9.019, *p* < 0.001, BF = 9.026e +9) and the +140 transitions (*t*(60) = 12.317, *p* < 0.001, BF = 1.245e +15), as well as in terms of the +20 transitions (*t*(60) = 9.000, *p* < 0.001, BF = 8.398e +9) but not the -20 transitions (*t*(60) = 1.290, *p* = 0.202, BF = 0.308). Specifically, compared to the Lookahead ‘rho’ model, participants sensitivities to the -140 and +140 transitions appear weaker and stronger, respectively, when pruning is taken into account (see Figure S1).


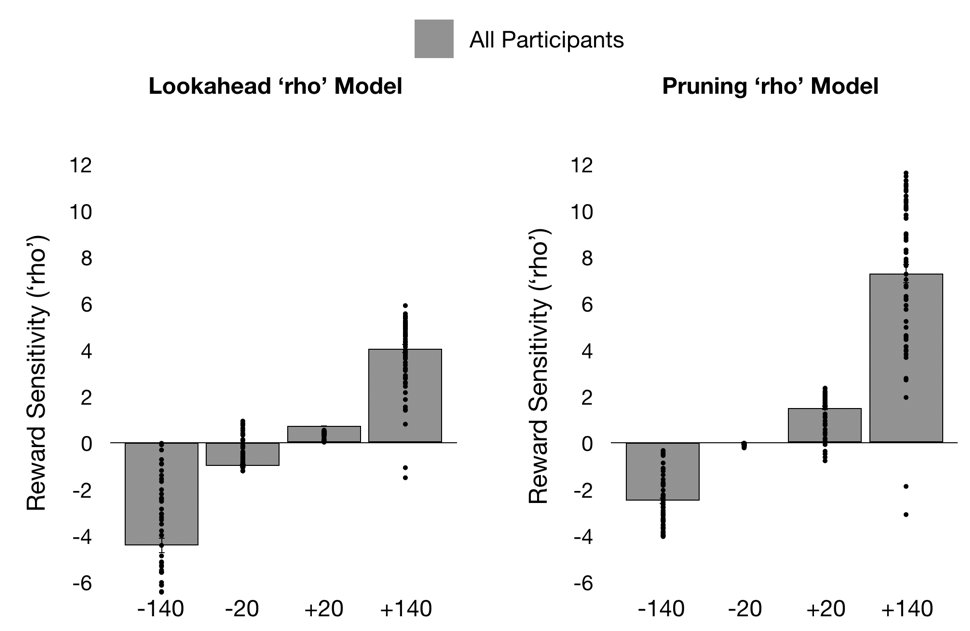


**Figure S1. Rho values for each of the four transition types in both the Lookahead ‘rho’ model (left) and the Pruning ‘rho’ model (right)**

Data pertaining to the number of depressive episodes experienced was collected from 18 of the 30 depressed individuals. Bivariate correlations were performed to determine whether the number of depressive episodes in these 18 participants was related to the pruning behaviours. Results revealed that the number of depressive episodes did not correlate with general pruning (r = -0.108, p = 0.671, BF = 0.286), specific pruning (r = 0.091, p = 0.720, BF = 0.282), or sensitivity to any of the four transition types (all ps > 0.206, all BFs < 0.553).

Bivariate correlations were performed to determine whether self-reported anhedonia on the BDI was related to pruning behaviours in depressed participants. Results revealed that such anhedonia was not related to either general pruning (r = -0.122, p = 0.365, BF = 0.247), or specific pruning (r = 0.157, p = 0.242, BF = 0.322). While there was no relationship between self-reported anhedonia and sensitivity to the -20 transition types (r = -0.055, p = 0.683, BF = 0.179), there were significant correlations between such anhedonia and sensitivity to the +140 transitions (r = -0.296, p = 0.025, BF = 1.901), +20 transitions (r = -0.270 , p = 0.042, BF = 1.236) and -140 transitions (r = 0.302, p = 0.023, BF = 2.088); however, when these analyses were Bonferroni-corrected for the four tests performed (i.e.one per transition type), none of these relationships remained significant.

T-tests revealed that there was no difference between those depressed individuals who received a diagnosis of generalized anxiety disorder (GAD) on the MINI and those who did not, in terms of general pruning (t(28) = 0.521, p = 0.607, BF = 0.387), specific pruning (t(28) = -0.172, p = 0.864, BF = 0.354) or sensitivity to any of the four transitions (all ps > 136, all BFs < 0.832).

Further, bivariate correlations were performed to determine whether scores from section ‘O’ of the MINI (i.e. the generalized anxiety disorder section) were related to pruning behaviours. Results revealed that these scores did not correlate with general pruning (r = -0.400, p = 0.100, BF = 1.026), specific pruning (r = 0.056, p = 0.826, BF = 0.298), or sensitivity to any of the four transition types (all ps > 0.358, all BFs < 0.653).

**Sensitivity analyses**

We also performed analyses of data from all 48 trials (as opposed to only the final 24 trials, as in Huys et al., 2012), to determine whether doing so altered the resultant parameter estimates and group comparisons.

Firstly, the parameters resulting from modelling all 48 trials are substantially correlated with the corresponding variables derived from modelling only the final 24 trials (γ_S_: r = 0.396, p = 0.003, BF = 21.061; γ_G_: r = 0.502, p < 0.001, BF = 67.641; sensitivity to the +140 transitions: r = 0.827, p < 0.001, BF = 334.013: sensitivity to the +20 transitions; r = 0.857, p < 0.001, BF = 408.015; sensitivity to the -20 transitions: r = 0.494, p < 0.001, BF = 198.377); sensitivity to the -140 transitions: r = 0.864, p < 0.001 , BF = 492.160; see figure S2 for an indication of these relationships).


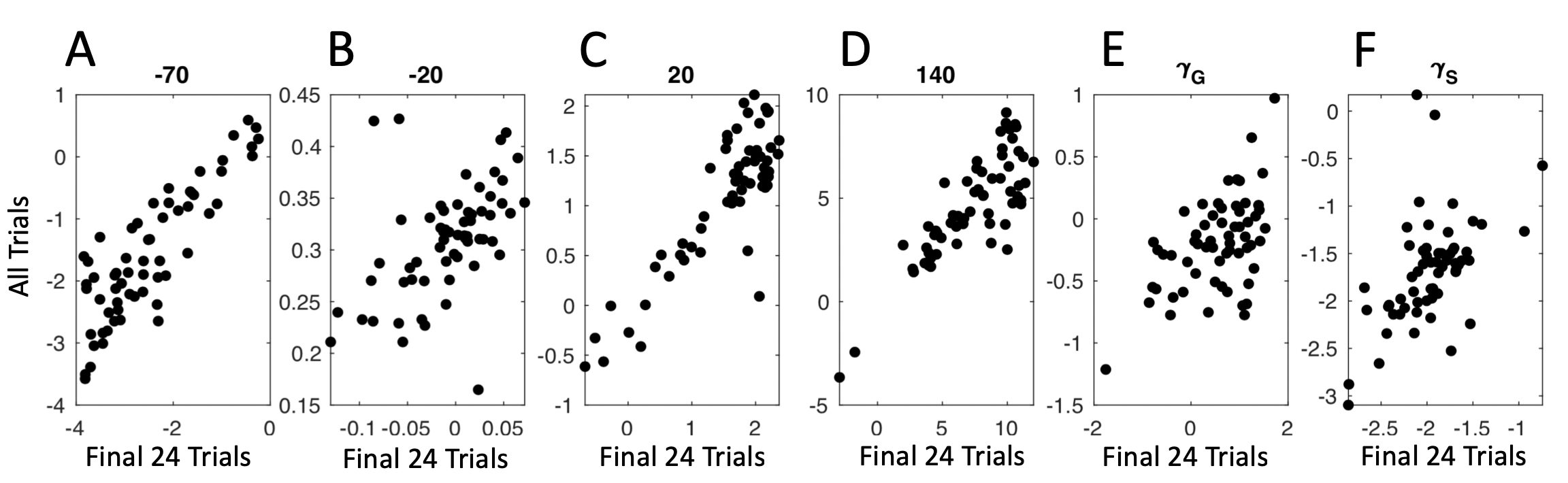


**Figure S2. Scatterplots depicting relationships between variables from the winning model when data from all trials were included in the analyses and when data from only the final 24 trials were included**

We also performed group comparisons to determine whether there were any meaningful differences between depressed and non-depressed participants in terms of the variables resulting from the winning model when applied to data from all 48 trials. There were no group differences in terms of γ_S_ (t(59) = 1.046, p = 0.300, BF = 0.413), γ_G_ (t(59) = -0.613, p = 0.542, BF = 0.305), sensitivity to the +140 transitions (t(59) = -0.042, p = 0.967, BF = 0.261), sensitivity to the +20 transitions (t(59) = -0.174, p = 0.862, BF = 0.264), sensitivity to the -20 transitions (t(59) = 1.014, p = 0.315, BF = 0.401) or sensitivity to the -140 transitions (t(59) = -0.430, p = 0.669, BF = 0.282).

Finally, in depressed participants, there were no significant or meaningful correlations between γ_S_, γ_G_ or any of the reward sensitivities with BDI scores (all ps > 0.165, all BFs < 0.418), the number of depressive episodes (all ps > 0.368, all BFs < 0.329), self-reported anhedonia (as quantified using the BDI; all ps > 0.415, all BFs < 0.259), STAI trait anxiety scores (all ps > 0.333, all BFs < 0.366), STAI state anxiety scores (all ps > 0.272, all BFs < 0.318), or scores from section ‘O’ of the MINI (i.e. those scores denoting presence and severity of GAD; (all ps > 0.178, all BFs < 0.408).
